# Risk Factors for Low Levels of Parathyroid Hormone after Surgery for Thyroid Cancer: A Single Center Study

**DOI:** 10.3390/jcm10184113

**Published:** 2021-09-12

**Authors:** Francesca Privitera, Rossella Gioco, Ileana Fazio, Alessio Volpicelli, Maria Teresa Cannizzaro, Salvatore Costa, Matteo Angelo Cannizzaro, Massimiliano Veroux

**Affiliations:** 1 Department of General Surgery, Azienda Ospedaliera Universitaria Policlinico San Marco, 95123 Catania, Italy; f.privitera05@gmail.com (F.P.); rosellagioco1992@gmail.com (R.G.); fazioileana@gmail.com (I.F.); alessiovolpicelli94321@gmail.com (A.V.); salvatore.costa@policlinico.unict.it (S.C.); cannizzaromatteoangelo@yahoo.it (M.A.C.); 2 Radiology Unit, Azienda Ospedaliera Universitaria Policlinico San Marco, 95123 Catania, Italy; maraterex@yahoo.it; 3 Department of Medical and Surgical Sciences and Advanced Technologies, University Hospital of Catania, 95123 Catania, Italy

**Keywords:** parathyroid hormone, hypocalcemia, thyroid cancer, incidental parathyroidectomy, parathyroid hormone, female, thyroidectomy, lobectomy, central neck dissection, lymph node

## Abstract

Background: Thyroidectomy is the definitive treatment for most patients with thyroid cancer. Hypoparathyroidism is the most frequent complication of thyroidectomy, and its pathogenesis is multifactorial. The aim of this study is to evaluate the patient- and surgical-related risk factors for hypoparathyroidism after surgery for thyroid cancer. Methods: In this retrospective study, patients referred to surgery for thyroid cancer from 2016 to 2019 were enrolled. Preoperative serum calcium and parathyroid hormone (PTH) and postoperative 24 h PTH and calcium levels were evaluated. Demographic data, type of surgery, incidence of hypoparathyroidism and hypocalcemia were recorded for all the patients. Patients were divided into two groups based on post-operative PTH levels (≤12 and >12 pg/mL). Results: A total of 189 patients were enrolled in this study. There were 146 women (87.3%) and 43 men (22.7%), with a mean age of 51.3 years. A total of 79 patients (41.7%) underwent a neck dissection. A total of 59 patients (31.1%) had a postoperative PTH level < 12 pg/mL. Female sex, neck dissection, the yield of lymph node dissection and incidental parathyroidectomy were significantly associated with postoperative hypoparathyroidism. Incidental parathyroidectomy was reported in 44 (23.2%) patients and was correlated with younger age (<40 years) and neck dissection. There was no difference in the rate of post-operative hypocalcemia between patients with incidental parathyroidectomy and those without. Conclusions: Young patients undergoing neck dissection and with incidental parathyroidectomy have the highest risk of postoperative hypoparathyroidism after surgery for thyroid cancer. However, a large proportion of patients without incidental parathyroidectomy may have temporary hypocalcemia, suggesting that impaired blood supply of parathyroid glands during their identification and dissection may play a relevant role.

## 1. Introduction

Thyroid cancer is the most common endocrine tumor and its incidence has significantly increased over the last three decades [1]. Although the prognosis of thyroid cancer is usually good, in most cases a surgical approach is required. Total thyroidectomy is usually recommended for patients with thyroid nodules in which thyroid cancer is suspected in order to improve survival and lower recurrence [2,3]. Total thyroidectomy is one of the most frequently performed endocrine surgical procedures, and it can lead to serious complications, including temporary or permanent cordal palsy or postoperative bleeding, although hypoparathyroidism is the most frequent complication [4,5,6]. Hypoparathyroidism with hypocalcemia may affect 3–49% of patients undergoing thyroid surgery [4,5,6,7,8,9,10,11,12,13,14]. Many risk factors have been associated with an increased incidence of post-operative hypocalcemia. Post-thyroidectomy hypocalcemia may arise from an incidental parathyroid removal, but the risk is also increased when a larger number of parathyroid glands are left in situ due to a compromised blood supply as a result of their dissection [6,9,13]; this risk could be further increased when a neck dissection is associated with a total thyroidectomy [6,8,9,10,11,13]. Parathyroid hormone (PTH) levels are the most frequently evaluated biochemical factor in the development of post-operative hypocalcemia, but the association between PTH levels and postoperative hypocalcemia has been reported with conflicting results [9,15,16]. Moreover, there is no consensus on the most useful predictive cut-off point for post-operative PTH levels and the appropriate time of PTH measurement after surgery: 1 h PTH level after skin closure may be predictive of postoperative hypocalcemia [6,8,9,10,11,13], while other studies suggested that a reduction in postoperative PTH of more than 44% [17] or more than 60% [18] is predictive of temporary biochemical hypocalcemia [7,14,19].

In this study, we evaluated the risk factors for low levels of PTH after surgery for thyroid cancer to identify the patient and surgical related factors that could have a significant correlation with the development of hypoparathyroidism.

## 2. Materials and Methods

All patients who were scheduled for surgical treatment for thyroid cancer between January 2016 and December 2019 were retrospectively reviewed. All patients were diagnosed with thyroid cancer based either on a fine needle aspiration or at final histological examination. Inclusion criteria included the following: normal biochemical results of calcium metabolism, normal serum albumin and total protein and normal thyroid, liver, and kidney functions.

Cases of completed thyroidectomy and patients with diseases or who were taking medications that affect calcium homeostasis were excluded. Each participant received and signed an informed consent form. Total extracapsular excision of the thyroid gland was performed on each patient by the same team of two surgeons. Central neck dissection, bilateral or ipsilateral lymphadenectomy, were performed on suspicious enlarged lymph nodes on the bilateral or ipsilateral sides. No prophylactic unilateral or bilateral lymphadenectomy was performed.

All parathyroids were visualized intraoperatively. In each case, the thyroid was devascularized by selective closure of distal branches of the thyroid arteries to spare the vascular supply of the parathyroids. Biochemical monitoring of mineral homeostasis included the following: serum calcium (Calcium Arsenazo III, Beckman Coulter, Inc., normal range: 8.1–10.2 mmol/L), phosphate (Inorganic Phosphorous, Beckman Coulter, Inc., normal range: 0.81–1.45 mmol/L) and PTH (Access Intact PTH Assay, Beckman Coulter, Inc., normal range: 12–88 pg/mL), measured pre-operatively and 24 h after surgery. Temporary postoperative hypocalcemia was considered for calcium serum levels lower than 8.0 mg/dL, measured 24 h after surgery. Hypoparathyroidism was considered for PTH levels < 12 pg/mL. Patients with biochemical hypocalcemia (<8 mg/dL) or with symptoms associated with serum calcium decrease were given calcium carbonate orally as well as calcium gluconate intravenously, when needed. Symptoms were monitored until discharge and thereafter on an outpatient basis. In case of symptoms of hypocalcemia or serum calcium < 8 mg/dL, an oral supplementation of calcium and calcitriol was administered until resolution of symptoms or normalization of serum calcium levels. Definitive hypocalcemia was defined as the need for treatment with calcium or calcitriol at 6 months after surgery.

Clinical and follow-up data were retrieved from our electronic database and included sex, age, preoperative serum calcium levels, preoperative serum PTH levels, surgical procedures (thyroidectomy/lobectomy) including neck dissection, number of retrieved lymph nodes, thyroid volume, incidental parathyroidectomy, post-operative calcium levels, post-operative PTH levels, definitive histological examination, presence of thyroiditis based on histopathology reports and surgical complications. Pathological staging was based on the tumor/nodes/metastases (TNM) system.

Pathology reports were reviewed for documentation of parathyroids identified in the specimen.

Patients were divided into two groups based on post-operative PTH levels (≤12 and >12 pg/mL). Thyroid volume was calculated through the measurements of the depth (d), the width (w) and the length (l) of each lobe, as reported at final histological examination. The volume is calculated by the ellipsoid formula:

V (mL) = 0.52 × d × w × 1 (cm)

Permanent hypoparathyroidism was considered at PTH levels ≤ 12 pg/mL or clinical symptoms of permanent hypocalcemia at 6-month follow-up [14]. Statistical data analysis was performed using SPSS (version 20.0; SPSS Inc., Chicago, IL, USA). Data are expressed as mean ± standard deviation (SD). To compare parametric variables, the Pearson chi-square test or Fisher’s exact test was used. To compare non-parametric variables, Student T test or Mann–Whitney U test was used. The difference between the two means was calculated using the Wilson test. Odds ratios (OR) were reported with 95% confidence interval (95% CI) and P values. The level of statistical significance was determined at *p* < 0.05.

## 3. Results

A total of 189 patients undergoing thyroid surgery for thyroid cancer between January 2016 and December 2019 were enrolled in this study. There were 146 women (87.3%) and 43 men (22.7%), with a mean age of 51.3 years (range, 19–75). Patients’ demographics, operative details, histological findings and postoperative events are reported in Table 1.

Most patients (183, 96.8%) underwent a total thyroidectomy, while six patients underwent a lobectomy (3.2%). A total of 79 patients (41.7%) underwent a neck dissection. Most patients were finally diagnosed with papillary cancer (163, 86.2%) or follicular cancer (44, 23.2%). Post-operative temporary hypocalcemia was present in 69 (36.5%) patients, while only five (2.6%) patients experienced a definitive hypocalcemia. Most patients with hypocalcemia presented with numbness and tingling in their fingertips, toes and the perioral region, while no patients presented convulsions.

A total of 59 patients (31.1%) had a postoperative PTH level < 12 pg/mL (Table 2). Younger female patients were at higher risk of low PTH levels after thyroid surgery. Hypoparathyroidism presented more frequently in female (37.3%) compared to male patients (12.7%, *p* < 0.001). Interestingly, the total thyroidectomy did not increase the risk of low PTH, but the neck dissection significantly increased the risk of parathyroid injury (OR = 2.56 (95% CI 1.36–4.82), *p* = 0.004), and patients with low postoperative PTH had a higher mean number of lymph nodes retrieved compared to patients with higher PTH levels (8.53 vs. 4.12, *p* = 0.005). Low levels of PTH were associated with an increased rate of incidental parathyroidectomy, which was present in 35.6% of the patients; this was associated with an OR of 2.44 (95% CI 1.22–4.88, *p* = 0.017). However, 25 patients (18.4%) with normal post-operative PTH levels had an incidental parathyroidectomy, suggesting that this is not the only factor contributing to the development of post-operative parathyroid dysfunction and hypocalcemia. Indeed, the number of parathyroid glands identified during surgery and auto-transplanted parathyroids did not correlate with the incidence of postoperative hypoparathyroidism.

While there was not a significant difference in pre-operative PTH levels between the two groups, mean postoperative PTH levels were significantly different (4.3 ± 3.63 pg/mL vs. 35.6 ± 17.5 pg/mL, *p* < 0.001). Overall, patients with postoperative hypocalcemia had 70.6% lower PTH levels compared with preoperative levels, and this difference was even more pronounced in patients with postoperative PTH levels < 12 pg/mL (92%). Histological type, cancer volume and thyroid volume did not correlate with post-operative PTH levels.

A total of 37 patients (63%) in the group of PTH < 12 pg/mL developed a temporary postoperative hypocalcemia, compared to 33 patients (25.4%, *p* < 0.01) in the group of PTH > 12 pg/mL. Interestingly, five patients (8%) with a postoperative PTH value < 12 pg/mL developed a definitive hypocalcemia, while no patient in the group with postoperative PTH levels > 12 pg/mL developed a definitive hypoparathyroidism. Female sex, age < 55 years and PTH levels < 12 pg/mL were predictive of postoperative temporary hypocalcemia, while the incidental parathyroidectomy did not increase the risk of hypocalcemia (Table 3).

All patients who developed a definitive hypocalcemia had a 1-day postoperative PTH level < 1 pg/mL, and a postoperative PTH level < 5 pg/mL was a strong predictive factor for definitive hypoparathyroidism (OR = 24.5 (95% CI 2.83–212.51, *p* < 0.0001). At the 6-month follow-up, serum calcium and PTH levels were similar among the two groups.

A subsequent analysis on risk factors for incidental parathyroidectomy was performed (Table 4). In 19 patients (10%), a parathyroid gland was auto-transplanted, of which only three developed a transient post-operative hypocalcemia. There was no significant difference in the incidence of incidental parathyroidectomy among patients < 55 years compared to those >55 years. However, when stratified for age, younger (<40 years) patients had the higher risk of having an incidental parathyroidectomy (RR 1.8 OR 2.2 (95% CI 1.02–4.88), compared to patients > 41 years (RR 0.9 OR 0.6 (95 CI 0.308–1.157). Patients who underwent neck dissection had an increased risk of incidental parathyroidectomy (OR 3.03 (95% CI 1.50–6.12, *p* < 0.001), with the risk increasing with the number of retrieved lymph nodes, being the highest for > 8 lymph-nodes (OR 1.7,95% CI 0.58–5.00, *p* = 0.044) retrieved. There was no significant correlation between the number of parathyroid glands identified during surgery and the risk of incidental parathyroidectomy. Patients with unintentional parathyroidectomy had significantly lower postoperative PTH levels (19.3± 19.2 vs. 27.3 ± 23 pg/mL, *p* = 0.03) and, although there was a higher incidence of postoperative biochemical hypocalcemia, this did not reach the statistical significance (45.4% vs. 34.4%, *p* = 0.194). There were no significant differences for gender or histological type.

## 4. Discussion

Hypoparathyroidism is the most common complication after thyroid surgery, but the true incidence is debatable due to the heterogeneity in classification and identification of this complication. A recent meta-analysis reported a median incidence of temporary and permanent hypoparathyroidism following thyroidectomy ranging from 19% to 38% and 0% to 3%, respectively, suggesting that a large number of patients undergoing thyroid surgery may suffer from this complication [13].

This study investigated the patient- and surgery-related risk factors associated with low postoperative PTH levels. After thyroidectomy, monitoring of PTH and serum calcium levels is mandatory for identifying the hypoparathyroidism before the development of severe and symptomatic hypocalcemia [20]. Because postoperative calcium levels may be confounded by prophylactic calcium and calcitriol administration, or by low preoperative vitamin D levels, many groups preferred the measuring of intraoperative or postoperative intact PTH levels drawn at various time points in the early post-thyroidectomy period [10,14,20]. A recent statement on hypoparathyroidism of the American Thyroid Association found that the timing of PTH measurements in published studies has ranged from 10 min to 24 h post-thyroidectomy [14], and that a postoperative PTH level < 15 pg/mL is usually predictive of hypocalcemia [6,8,9,10,11,13,20,21]. However, serum PTH levels may remain stable within the first days after thyroidectomy and day 1 PTH levels may be accurate enough to predict hypocalcemia and direct the initiation of calcium supplementation [4,21].

In our study, postoperative hypocalcemia developed in 36.5% of patients, while a total of 59 patients (31.1 %) had a postoperative PTH level < 12 pg/mL. PTH levels may be a significant predictive factor for post-operative hypoparathyroidism and hypocalcemia: among the 37 patients with PTH < 12 pg/mL who developed a temporary postoperative hypocalcemia, five patients (8%) developed a definitive hypocalcemia, while no patients with hypocalcemia and PTH levels > 12 pg/mL developed a definitive hypocalcemia.

Postoperative PTH levels are significantly related to postoperative hypocalcemia [22], and a recent systematic review showed that patients with a decrease in post-operative PTH had a 69–100% chance of developing temporary hypocalcemia [13]. Moreover, the accuracy of an absolute PTH level to predict temporary hypocalcemia ranges from 34% to 100%, while the accuracy for a change in PTH ranges from 72% to 100%; however, the development of hypocalcemia despite a normal PTH level is up to 54% for an absolute PTH value and up to 50% for a percentage change in PTH, suggesting that even patients with a normal PTH can develop hypocalcemia [13]. This assumption was further demonstrated by Del Rio et al. [6] who showed that, among the 101 patients presenting with hypocalcemia (serum calcium < 7.5mg/dL) beyond postoperative day 1, only 49 had PTH values less than 12 pg/mL, whereas the others 52 patients had PTH values within the normal range; additionally, there was no statistically significant difference in absolute PTH values in patients with hypocalcemia compared with patients with eucalcemia [6].

This was also confirmed in our study where, although a higher incidence of post-operative hypocalcemia (serum calcium < 8 mg/dL) was observed in patients with a lower postoperative PTH level (<12 pg/mL), there was no significant difference of PTH levels between patients with postoperative hypocalcemia and patients with normocalcemia; this suggests that low PTH levels, although potentially predictive of postoperative temporary hypocalcemia, do not indicate an absolute risk of hypocalcemia, and similarly, a normal PTH value does not guarantee normocalcemia.

The mechanism of hypoparathyroidism after thyroidectomy has not been fully elucidated and is likely to be multifactorial, including surgical technique, parathyroid injury, patient gender, incidental parathyroidectomy and neck dissection [14,20].

Age < 40 years was found to be significantly associated with hypoparathyroidism. In literature, there are conflicting data about the correlation between post-operative hypoparathyroidism and patient age: while temporary hypocalcemia may be associated either with advanced age [16] or younger age [23,24], most studies found no significant association with age [2,10,13]. More recently, a retrospective study on 278 Chinese patients found a significant association between age and postoperative hypocalcemia [5], while Del Rio et al. [6] did not find such association among 2108 patients undergoing thyroid surgery for benign and malignant diseases.

Many studies tried to find an explanation to female predisposition to post-thyroidectomy hypocalcemia [6] and, although the specific mechanism is not certain, the gender disparity may be related to effects of sexual steroids on PTH secretion [25,26]. Female patients were at higher risk of developing postoperative hypoparathyroidism (53/142, 37.3%) compared to male patients (6/47, 12.7%, *p* < 0.001). Female sex and age were found to be significant risk factors for postoperative hypocalcemia with conflicting results: in their retrospective study, Karadeniz and Akcay [27], found that young age (<28.5 years old) and female sex were risk factors for post-operative hypocalcemia; in contrast, Algarni’s retrospective analysis [28] found no significant correlation with female sex, probably because of the small sample size of the study (40 patients).

The extent of surgery may influence the rate of postoperative hypoparathyroidism. The number of parathyroids glands identified during surgery and auto-transplanted parathyroids did not influence the rate of post-operative hypocalcemia, as reported in many studies [9]. It should be noted that in patients with thyroid cancer, parathyroid glands may be not easily identified since they could be confounded with enlarged lymph-nodes or with the fat tissue surrounding the thyroid; this could partially explain the higher rate of incidental parathyroidectomy in patients with thyroid cancer.

In our study, patients who underwent neck dissection had an increased risk of parathyroid injury. Moreover, patients with low post-operative PTH levels had a higher mean number of lymph-nodes retrieved compared to patients with higher PTH levels. Total thyroidectomy is seen to have an equivocal association with symptomatic hypocalcemia, with little evidence suggesting an association with either temporary or permanent hypocalcemia [10]. However, neck dissection demonstrates a significant association with hypoparathyroidism [5,16,23,26], but a recent meta-analysis demonstrated that the addition of neck dissection to total thyroidectomy shows an association only with symptomatic and permanent hypocalcemia, but not with temporary biochemical hypocalcemia [10].

No significant correlation was found between histological type, cancer volume and thyroid volume and postoperative hypoparathyroidism, as reported in other studies [27]. In contrast, Mo et al. [4], in their study investigating the risk for temporary hypocalcemia in 176 patients undergoing total thyroidectomy for papillary thyroid carcinoma, found that tumor diameter was a risk factor for temporary hypocalcemia in female patients, while histological diagnosis of papillary cancer may be related to an increased incidence of postoperative hypocalcemia [26].

Incidental parathyroidectomy was present in 44 (23.2%) patients, and it correlated significantly with low postoperative PTH levels. This was consistent with data reported in literature, where incidental parathyroidectomy was identified in 4%–28% of thyroid specimens [21,27,29,30,31,32,33]. In this study, incidental parathyroidectomy was correlated with younger age (<40 years) and with neck dissection with higher lymph-node yield, while total thyroidectomy and histological findings did not increase the incidence of incidental parathyroidectomy. Malignancy and neck dissection, together with the surgeon’s experience have been identified as the strongest risk factors associated with incidental parathyroidectomy [26,27,29,30,31,32,33]. In their study, Barrios et al. [30], among 1114 thyroidectomies and 396 concurrent central neck dissections performed across seven surgeons, found that central neck dissection, either prophylactic or therapeutic, but not the yield of lymphadenectomy, increased the risk of incidental parathyroidectomy (OR 2.68 and 4.44, respectively). In contrast, the surgeon’s experience had a protective role, suggesting that high-volume surgeons could safely perform more extensive central neck dissections with lower incidences of complications [30]. The extent of thyroid surgery is not necessarily associated with increased risk of incidental parathyroidectomy [26,27,29,30,31,32,33], as reported in our experience. Interestingly, incidental parathyroidectomy was associated with a higher risk of temporary hypoparathyroidism and with permanent hypocalcemia, although not statistically significant, but not with temporary hypocalcemia. This apparent paradox may be correlated to the function of the remaining parathyroid glands [34]. However, the 26.8% and the 34.4% of patients without incidental parathyroidectomy experienced temporary postoperative hypoparathyroidism and hypocalcemia, respectively, suggesting that extensive identification and dissection of parathyroids may compromise their blood supply and, therefore, their function [9,30,35].

The main limitations of this study are the retrospective nature and the relatively small sample size. However, surgical procedures were performed by the same surgical team in a high-volume center, and this could reduce the bias caused by different surgeon experience.

In conclusion, surgery for thyroid cancer may be associated to an increased risk of postoperative hypoparathyroidism and hypocalcemia. While neck dissection and incidental parathyroidectomy may increase the rate of postoperative hypoparathyroidism, a large proportion of patients without incidental parathyroidectomy may experience postoperative hypocalcemia, suggesting that a careful surgical technique is recommended for reducing the risk of post-operative complications.

## Figures and Tables

**Table 1:** Patients’ characteristics.

Characteristic	*N* (%)
**Age**	
Male	43 (22.8)
Female	146 (77.2)
**Mean age (years)**	51.3 ± 22.4
**Surgical Procedure**	
Total Thyroidectomy	183 (96.8)
Lobectomy	6 (3.2)
**Histological type**	
Papillary	163 (86.2)
Follicular	44 (23.2)
Other (medullary, anaplastic, rare tumors)	7 (3.7)
**TNM Classification**	
T1	130 (68.7)
T2	10 (5.3)
T3	48 (25.6)
T4	1 (5.4)
Lymph-node metastasis (N+)	12 (6.4)
**Unintentional parathyroidectomy**	44 (23.3)
Portion of parathyroid	12 (26.3)
One Parathyroid	32 (72.7)
**Parathyroid glands identified during surgery**	
0	3 (1.5)
1	6 (3.2)
2	45 (23.9)
3	81(42.9)
4	54 (28.5)
**Auto-transplanted parathyroids**	20 (10.5)
**Post-operative hypocalcemia**	69 (36.5%)
**Definitive Hypocalcemia**	5 (2.6)

**Table 2:** Risk factors for low level of postoperative PTH.

Characteristics	PTH < 12 pg/mL *N* (%)	PTH > 12 pg/mL *N* (%)	*p* Value
**Patients**	59 (31.1)	130 (68.9)	
**Age (mean, years)**	48.4 ± 12.2	53.4 ± 11.8	**0.011**
Age Groups			
<40	16 (27.1)	23 (17.7)	**0.008**
41–55	29 (49.1)	45 (34.6)	0.849
>55	14 (23.7)	62 (47.6)	0.754
**Sex**			
Male	6 (10.2)	41 (31.6)	
Female	53 (89.8)	89 (68.4)	**<0.001**
**Surgical Procedure**			
Total Thyroidectomy	59 (100)	124 (95)	0.898
Lobectomy	0	6 (5)	
**Neck dissection**	34 (58)	45 (35)	**0.004**
**Central neck dissection**	15 (25.4)	20 (15.3)	0.622
**Unilateral Lymphadenectomy**	14 (23.7)	20 (15.3)	0.532
**Bilateral lymphadenectomy**	5 (8.4)	5 (3.8)	0.455
**PTH levels (mean)**			
Preoperative	53.6 ± 26.9	53.1 ± 20.5	0.884
Postoperative	4.3 ± 3.63	35.6 ± 17.5	**<0.001**
**Incidental Parathyroidectomy**			
No	38 (64.4)	107 (82.4)	
Yes	21 (35.6)	23 (17.6)	**<0.001**
**Auto-transplanted parathyroids**	3 (6.7)	16 (11.5)	0.312
**Parathyroid glands identified during surgery**			
0	1 (1.6)	2 (1.5)	0.936
1	2 (3.4)	4 (3)	0.912
2	15 (25.4)	30 (23)	0.726
3	21 (35.6)	60 (46.3)	0.173
4	20 (34)	34 (26.2)	0.275
**Histological type**			
Papillary	55 (93.2)	108 (83)	0.832
Follicular	11 (18.6)	33 (25.3)	0.651
Other (medullary, anaplastic, rare tumors)	0	7 (5.3)	0.821
**Thyroid volume (mean, cm^2^)**	23.43 ± 26.52	24.9 ± 26.4	0.724
**Cancer volume (mean, cm)**	0.93 ± 0.6	1.02 ± 0.9	0.654
**Number of retrieved lymph nodes (mean)**	8.53	4.12	**0.005**
**Postoperative Hypocalcemia**			
Temporary	37 (63)	33 (25.4)	**<0.05**
Definitive	5 (8)	0	
Preoperative/postoperative PTH levels ratio	92%	49.5%	**<0.05**
**TNM Classification**			
**T1**	36 (61)	94 (71.1)	0.120
**T2**	3 (5)	7 (5.4)	0.622
**T3**	20 (34)	28 (21.5)	0.070
**T4**	0	1 (0.9)	
**N+**	36(61)	54 (41.5)	**0.012**
**6-month Postoperative serum calcium (mean, g/dL)**	8.8 ± 0.53	9.3 ± 0.42	0.643
**6-month postoperative PTH level (mean, pg/mL)**	18.22 ± 8.34	26.3 ± 10.4	0.138

**Table 3:** Risk factors for temporary hypocalcemia.

Characteristics	OR	95% CI	*p* Value
**Sex**			
Male	1		
Female	4.06	1.618–10.228	**<0.05**
**Age (ys)**			
<40	1.73	0.83–3.59	**<0.01**
41–55	1.82	0.97–3.41	**<0.05**
>55	1		
**PTH < 12 pg/mL**	4.94	2.55–9.55	**<0.001**
**Incidental parathyroidectomy**	0.63	0.31–1.25	0.186

**Table 4:** Risk factors for un-intentional parathyroidectomy.

Characteristics	Unintentional Parathyroidectomy	No Parathyroidectomy	
	*N* (%)	*N*(%)	*p* Value
**Patients**	44(23.2)	145 (76.7)	
**Sex**			
Male	11 (25)	35 (24.2)	0.743
Female	33 (75)	110 (75.8)	0.896
**Age (years, %)**	50.1 ± 14.1	51.7 ± 11.9	0.451
<55	26 (59)	83 (57.2)	
>55	18 (41)	62 (42.8)	0.827
Age Groups			
<40	13 (29.5)	21 (14.5)	**0.015**
41–55	13 (29.5)	63 (43.4)	0.123
>55	18 (41)	61 (42.1)	0.091
**Total thyroidectomy/lobectomy**	43 (97.7)	140 (96.6)	0.833
**Lobectomy**	1 (2.3)	5 (4.6)	0.901
**Neck dissection**	28 (63.6)	51 (35.1)	**0.001**
**Number of retrieved lymph nodes**			
**<4**	15	30	OR 1 (95% CI 0.39–2.78)
**5–8**	2	10	OR 0.3 (95% CI 0.07–1.76)
**>8**	8	11	OR 1.7 (95% CI 0.58–5.00)
**Parathyroid glands identified during surgery**			
0	1 (2.2)	2 (1.3)	0.674
1	2 (4.6)	4 (2.8)	0.555
2	13 (29.5)	32 (22)	0.307
3	16 (36.3)	65 (44.8)	0.322
4	12 (27.2)	42 (28.9)	0.215
**Preoperative PTH (mean, pg/mL)**	55.9 ± 27	52.5 ± 21.3	0.396
**Postoperative PTH (mean, pg/mL)**	19.3 ± 19.2	27.3 ± 23	**0.03**
**PTH < 12 pg/mL**	21 (47.7)	39 (26.8)	**0.009**
**Temporary Hypocalcemia < 8 mg/dL**	20 (45.4)	50 (34.4)	0.194
**Definitive Hypocalcemia**	4 (10)	1 (2)	0.135
**Underlying disease**			
Papillary	37 (84.1)	126 (86.9)	0.626
Follicular	12 (27.3)	32 (22.1)	0.435
Hashimoto thyroiditis	6 (13.6)	31 (21.4)	0.201
Others	3 (6.8)	4 (6.8)	0.832
**Thyroid volume** (mean, cm^2^)	16.4 ± 8.45	26.5 ± 28.5	**0.002**
**Postoperative serum calcium (mean, mg/dL)**	8.1 ± 0.65	8.3 ± 0.6	0.07
**6-month Postoperative serum calcium (mean, g/dL)**	9.1 ± 0.55	9.6 ± 0.35	0.845

Bold of numbers was for those statistically significant.

## Data Availability

It is possible for de-identified data to be made available upon reasonable request.

## References

[1] Davies L., Welch H.G. (2006). Increasing incidence of thyroid cancer in the United States, 1973–2002. JAMA.

[2] Carling T., Udelsman R. (2014). Thyroid cancer. Annu. Rev. Med..

[3] Wilson C. (2011). Surgery: Benign thyroid disease-total or subtotal thyroidectomy?. Nat. Rev. Endocrinol..

[4] Mo K., Shang J., Wang K., Gu J., Wang P., Nie X., Wang W. (2020). Parathyroid Hormone Reduction Predicts Transient Hypocalcemia after Total Thyroidectomy: A Single-Center Prospective Study. Int. J. Endocrinol..

[5] Wang Y.H., Bhandari A., Yang F., Zhang W., Xue L.J., Liu H.G., Zhang X.H., Chen C.Z. (2017). Risk factors for hypocalcemia and hypoparathyroidism following thyroidectomy: A retrospective Chinese population study. Cancer Manag. Res..

[6] Del Rio P., Rossini M., Montana C.M., Viani L., Pedrazzi G., Loderer T., Cozzani F. (2019). Postoperative hypocalcemia: Analysis of factors influencing early hypocalcemia development following thyroid surgery. BMC Surg..

[7] Puzziello A., Gervasi R., Orlando G., Innaro N., Vitale M., Sacco R. (2015). Hypocalcaemia after total thyroidectomy: Could intact parathyroid hormone be a predictive factor for transient postoperative hypocalcemia?. Surgery.

[8] De Carvalho G.B., Diamantino L.R., Schiaveto L.F., Forster C.H.Q., Shiguemori É.H., Hirata D., Kohler H.F., Lira R.B., Vartanian J.G., Matieli J.E. (2021). Identification of secondary predictive factors for acute hypocalcemia following thyroidectomy in patients with low postoperative parathyroid hormone levels without overt calcium deficiency: A cohort study. Am. J. Otolaryngol..

[9] McMurran A.E.L., Blundell R., Kim V. (2020). Predictors of post-thyroidectomy hypocalcaemia: A systematic and narrative review. J. Laryngol. Otol..

[10] Wang X., Zhu J., Liu F., Gong Y., Li Z. (2017). Preoperative vitamin D deficiency and postoperative hypocalcemia in thyroid cancer patients undergoing total thyroidectomy plus central compartment neck dissection. Oncotarget.

[11] Godlewska P., Benke M., Stachlewska-Nasfeter E., Gałczyński J., Puła B., Dedecjus M. (2020). Risk factors of permanent hypoparathyroidism after total thyroidectomy and central neck dissection for papillary thyroid cancer: A prospective study. Endokrynol. Pol..

[12] Azadbakht M., Emadi-Jamali S.M., Azadbakht S. (2021). Hypocalcemia following total and subtotal thyroidectomy and associated factors. Ann. Med. Surg..

[13] Edafe O., Antakia R., Laskar N., Uttley L., Balasubramanian S.P. (2014). Systematic review and meta-analysis of predictors of post-thyroidectomy hypocalcaemia. Br. J. Surg..

[14] Orloff L.A., Wiseman S.M., Bernet V.J., Fahey T.J., Shaha A.R., Shindo M.L., Snyder S.K., Stack B.C., Sunwoo J.B., Wang M.B. (2018). American Thyroid Association Statement on Postoperative Hypoparathyroidism: Diagnosis, Prevention, and Management in Adults. Thyroid.

[15] Calò P.G., Conzo G., Raffaelli M., Medas F., Gambardella C., de Crea C., Gordini L., Patrone R., Sessa L., Erdas E. (2017). Total thyroidectomy alone versus ipsilateral versus bilateral prophylactic central neck dissection in clinically node-negative differentiated thyroid carcinoma. A retrospective multicenter study. Eur. J. Surg. Oncol..

[16] Docimo G., Ruggiero R., Casalino G., Del Genio G., Docimo L., Tolone S. (2017). Risk factors for postoperative hypocalcemia. Updates Surg..

[17] Chapman D.B., French C.C., Leng X., Browne J.D., Waltonen J.D., Sullivan C.A. (2012). Parathyroid hormone early percent change: An individualized approach to predict postthyroidectomy hypocalcemia. Am. J. Otolaryngol..

[18] Lecerf P., Orry D., Perrodeau E., Lhommet C., Charretier C., Mor C., Valat C., Bourlier P., de Calan L. (2012). Parathyroid hormone decline 4 hours after total thyroidectomy accurately predicts hypocalcemia. Surgery.

[19] Caglià P., Puglisi S., Buffone A., Bianco S.L., Okatyeva V., Veroux M., Cannizzaro M.A. (2017). Post-thyroidectomy hypoparathyroidism, what should we keep in mind?. Ann. Ital. Chir..

[20] Gafni R.I., Collins M.T. (2019). Hypoparathyroidism. N. Engl. J. Med..

[21] Selberherr A., Scheuba C., Riss P., Niederle B. (2015). Postoperative hypoparathyroidism after thyroidectomy: Efficient and cost-effective diagnosis and treatment. Surgery.

[22] Eismontas V., Slepavicius A., Janusonis V., Zeromskas P., Beisa V., Strupas K., Dambrauskas Z., Gulbinas A., Martinkenas A. (2018). Predictors of postoperative hypocalcemia occurring after a total thyroidectomy: Results of prospective multicenter study. BMC. Surg..

[23] White M.G., James B.C., Nocon C., Nagar S., Kaplan E.L., Angelos P., Grogan R.H. (2016). One-hour PTH after thyroidectomy predicts symptomatic hypocalcemia. J. Surg. Res..

[24] Kaleva A.I., Hone R.W., Tikka T., Al-Lami A., Balfour A., Nixon I.J. (2017). Predicting hypocalcaemia post-thyroidectomy: A retrospective audit of results compared to a previously published nomogram in 64 patients treated at a district general hospital. Clin. Otolaryngol..

[25] Sands N.B., Payne R.J., Côté V., Hier M.P., Black M.J., Tamilia M. (2011). Female gender as a risk factor for transient post-thyroidectomy hypocalcemia. Otolaryngol. Head Neck Surg..

[26] Coimbra C., Monteiro F., Oliveira P., Ribeiro L., de Almeida M.G., Condé A. (2017). Hypoparathyroidism following thyroidectomy: Predictive factors. Acta. Otorrinolaringol. Esp..

[27] Karadeniz E., Akcay M.N. (2019). Risk Factors of Incidental Parathyroidectomy and its Relationship with Hypocalcemia after Thyroidectomy: A Retrospective Study. Cureus.

[28] Algarni M., Alzahrani R., Dionigi G., Hadi A.H., AlSubayea H. (2017). Parathyroid hormone and serum calcium levels measurements as predictors of postoperative hypocalcemia in total thyroidectomy. Gland Surg..

[29] Du W., Fang Q., Zhang X., Cui M., Zhao M., Lou W. (2017). Unintentional parathyroidectomy during total thyroidectomy surgery: A single surgeon’s experience. Medicine.

[30] Barrios L., Shafqat I., Alam U., Ali N., Patio C., Filarski C.F., Bankston H., Mallen-St Clair J., Luu M., Zumsteg Z.S. (2021). Incidental parathyroidectomy in thyroidectomy and central neck dissection. Surgery.

[31] Sitges-Serra A., Gallego-Otaegui L., Suarez S., Lorente-Poch L., Munne A., Sancho J.J. (2017). Inadvertent parathyroidectomy during total thyroidectomy and central neck dissection for papillary thyroid carcinoma. Surgery.

[32] Bai B., Chen Z., Chen W. (2018). Risk factors and outcomes of incidental parathyroidectomy in thyroidectomy: A systematic review and meta-analysis. PLoS ONE.

[33] Lin Y.S., Hsueh C., Wu H.Y., Yu M.C., Chao T.C. (2017). Incidental parathyroidectomy during thyroidectomy increases the risk of postoperative hypocalcemia. Laryngoscope.

[34] Promberger R., Ott J., Kober F., Karik M., Freissmuth M., Hermann M. (2011). Normal parathyroid hormone levels do not exclude permanent hypoparathyroidism after thyroidectomy. Thyroid.

[35] Chew C., Li R., Ng M.K., Chan S.T.F., Fleming B. (2018). Incidental parathyroidectomy during total thyroidectomy is not a direct cause of post-operative hypocalcaemia. ANZ J. Surg..

